# Nephroprotective and Anti-Diabetic Potential of *Beta vulgaris* L. Root (Beetroot) Methanolic Extract in a Rat Model of Type 2 Diabetes Mellitus

**DOI:** 10.3390/medicina60030394

**Published:** 2024-02-26

**Authors:** Laila Naif Al-Harbi, Ghedeir M. Alshammari, Ghalia Shamlan, Manal Abdulaziz Binobead, Sahar Abdulaziz AlSedairy, Doha M. Al-Nouri, Shaista Arzoo, Mohammed Abdo Yahya

**Affiliations:** Department of Food Science and Nutrition, College of Food Science and Agriculture, King Saud University, Riyadh 11451, Saudi Arabia; aghedeir@ksu.edu.sa (G.M.A.); shamlana@ksu.edu.sa (G.S.); mbinobead@ksu.edu.sa (M.A.B.); ssudairy@ksu.edu.sa (S.A.A.); dr_nouri@ksu.edu.sa (D.M.A.-N.); sarzoo@ksu.edu.sa (S.A.); mabdo@ksu.edu.sa (M.A.Y.)

**Keywords:** nephroprotective, *Beta vulgaris* L., streptozotocin, fasting glucose, anti-inflammatory, antioxidant

## Abstract

*Background and Objectives*: Diabetes mellitus is a chronic metabolic disease associated with several complications, including that of kidney disease. Plant-based dietary products have shown promise in mitigating these effects to improve kidney function and prevent tissue damage. This study assessed the possible favorable effects of beetroot extract (BE) in improving kidney function and preventing tissue damage in diabetic rats. *Materials and Methods*: Type 2 diabetes mellitus (T2DM) was induced using a low dose of streptozotocin (STZ). Both control and rats with pre-established T2DM were divided into six groups (each consisting of eight rats). All treatments were given by gavage and continued for 12 weeks. Fasting blood glucose levels, serum fasting insulin levels, Homeostatic Model Assessment for Insulin Resistance (HOMA-IR), serum triglycerides, cholesterol, low-density lipoprotein-cholesterol, serum and urinary albumin, and creatinine and urea levels were measured. Apart from this, glutathione, malondialdehyde, superoxide dismutase, tumor necrosis factor-α, and interleukine-6 in the kidney homogenates of all groups of rats were measured, and the histopathological evaluation of the kidney was also performed. *Results*: It was observed that treatment with BE increased body weight significantly (*p* ≤ 0.05) to be similar to that of control groups. Fasting glucose, insulin, HOMA-IR levels, and lipid profile in the plasma of the pre-established T2DM rats groups decreased to *p* ≤ 0.05 in the BE-treated rats as the BE concentration increased. Treatment with BE also improved the renal levels of oxidative stress and inflammatory markers, urinary albumin, and serum creatinine and urea levels. Unlike all other groups, only the kidney tissues of the T2DM + BE (500 mg/kg) rats group showed normal kidney tissue structure, which appears to be similar to those found in the kidney tissues of the control rats groups. *Conclusion*: we found that streptozotocin administration disturbed markers of kidney dysfunction. However, *Beta vulgaris* L. root extract reversed these changes through antioxidant, anti-inflammatory, and antiapoptotic mechanisms.

## 1. Introduction

Recent advances have shown the significant role of inorganic nitrates in mediating cellular processes [1]. In general, inorganic nitrates mediate their effect via nitric oxide (NO), which regulates vascular tone, neurotransmission, mitochondrial respiration, and skeletal muscle contractile function [1]. NO may be produced via the canonical NO synthase-catalyzed oxidation of L-arginine and also by the sequential reduction of nitrate to nitrite and then to NO [1]. The body’s nitrate stores can be augmented by the ingestion of nitrate-rich foods [2]. Due to its abundant nitrate content, beetroot (*Beta vulgaris*) intake is well recognized to impart beneficial health effects such as lowering blood pressure, cognitive impairment, and risk of developing cardiovascular and other metabolic diseases [3,4]. In this regard, accumulating data are showing potent hypoglycemic and hypolipidemic effects of beetroot extracts (BE) in diabetic and obese individuals or animal models by acting through several mechanisms on the gut, liver, and adipose tissue [5,6,7,8]. Also, beetroot consumption is found to protect the kidneys and improve renal health in a variety of conditions [9]. Indeed, beetroot extract (BE) significantly ameliorated silver nanoparticle-induced nephrotoxicity by modulating oxidative stress and improving renal function [10]. Also, treatment with BE alleviated renal oxidative and inflammatory damage in rodents intoxicated with organophosphorus pesticides and chlorpyrifos [11]. Likewise, BE augmented kidney antioxidant levels and improved apoptotic and inflammatory parameters in rats against gentamycin-induced renal toxicity [12,13].

On the other hand, diabetes mellitus (DM) is a chronic metabolic disease associated with several complications [14]. Diabetic kidney disease (DKD) is a major microvascular complication that appears early in diabetic patients [15]. It has been estimated that the worldwide prevalence of diabetes will reach 642 million people by 2040 and, of these 40% will develop chronic kidney disease (CKD) [16]. Elevated blood glucose, hyperlipidemia, and alterations in hemodynamic parameters are known to promote kidney damage in diabetic animals and individuals by increasing the generation of reactive oxygen species (ROS), inflammatory cytokines, and apoptotic markers [17]. Within this view, it was shown that high glucose levels induce damage in the renal components, nephrons, or kidney tissue by overproduction of ROS through activating NADPH oxidase and other damaging pathways such as the polyol pathway, hexosamine pathway, or protein kinase C (PKC) pathway or through the generation of ROS [18]. Furthermore, hyperglycemia, insulin resistance, and compensatory hyperinsulinemia independently cause endothelial dysfunction by promoting some intracellular mechanisms, such as increased ROS production, PKCs, and advanced glycation end-products (AGE)-induced pro-inflammatory signaling [19]. Renal fibrosis, the final common pathway in the pathophysiology of DKD, is caused by at least renal hemodynamic changes, ischemia, and glucose metabolism abnormalities-associated oxidative stress increases, overactive renin–angiotensin–aldosterone system (RAAS) and inflammatory processes [19].

On the other hand, plant-based dietary products (i.e., phytochemicals) are shown to be an effective and promising therapy to mitigate these effects, improve kidney function, and prevent tissue damage [20]. The effect of BE on diabetes-induced kidney disease has not been investigated. In this study, we assessed the possible favorable effects of BE in improving kidney function and preventing tissue damage in a rat model of type 2 DM (T2DM). Further, we examined the possible underlying mechanisms that facilitate these effects.

## 2. Materials and Methods

### 2.1. Animals

In this study, a total of forty Wistar albino male rats (weighing 220–240 gm/12 weeks old) were obtained from the Experimental Animal Care Center, KSU. They were housed in individual cages under homogenous conditions (22 ± 5 °C, 55 ± 5%, and 12 h light/dark cycles), and water was provided ad libitum.

### 2.2. Animal Diets

The standard diet, consisting of 3.82 kcal/g (10%) fat; and the high-fat diet (HFD), containing 4.7 kcal/g (45%) fat were purchased from Research Diets Inc. (New Brunswick, NJ, USA) (Cat # D12450B and Cat # D12451, respectively).

### 2.3. Preparation of Beetroot Stems Methanol Extract

Fresh beetroot plants were procured from the local markets in Saudi Arabia. To begin with, the plant was washed, cleaned, and stripped of its skin, and the pulp was extracted and cut into smaller pieces and dried at 40 °C. Following this, the dried pulp was ground and soaked for 2 days in methanol at a 1:10 (*w*/*v*) ratio, which was then filtered, dried, and lyophilized before being stored at −80 °C for further analysis. At this time, the extract was dissolved in 5% carboxymethyl cellulose (CMC) as a carrier. This extract was named BE.

### 2.4. GC–MS analysis of Beta vulgaris (L.) Pulp Extract

The chemical constituents of BE were identified by gas chromatography and a mass spectrophotometer (PerkinElmer, Waltham, MA, USA). The temperature program was fixed to 40 °C, followed by a 2 min hold, and then raised to 200 °C at a rate of 5 °C min^−1^, which was also then put on hold for 2 min. From 200 °C, the temperature was raised by 5 °C min^−1^ to 300 °C and held for another 2 min. Chemical composition was determined by comparing the mass spectra obtained from the National Institute of Standard and Technology Spectral (NIST) library.

### 2.5. Establishment of T2DM

Type 2 diabetes mellitus was induced using a low dose of streptozotocin (STZ) and HFD feeding, as discussed previously [8,21]. Briefly, STZ (Cat # sc-200719A, Santa Cruz Biotechnology, Dallas, TX, USA) was prepared freshly in 0.1 M sodium citrate buffer (pH = 5.5). During the first two weeks, the rats received only HFD, followed by a single low dose of STZ (35 mg/kg) on day 14, and then continued on HFD for another three weeks. In the 36th-day experimental protocol of induction of diabetes in the animal models, blood glucose levels were measured using a glucometer, and those with a fasting glucose > 250 mg/dL were considered to have T2DM and were included in the experiment.

### 2.6. Experimental groups

Both control rats and rats with pre-established T2DM were divided into 6 groups (each of 8 rats) as follows:

(1) Control rats: fed the standard diets and administered 5% carboxymethyl cellulose (CMC) as a vehicle.
(2) Control + BE (250 mg/kg)-treated groups: fed the standard diet and orally administered BE extract (250 mg/kg/day).
(3) Control + BE (500 mg/kg)-treated groups: fed a standard diet and orally treated with BE extract alone (500 mg/kg/day).
(4) T2DM model group: rats with pre-established T2DM which fed HFD and orally administered 5% CMC.
(5) T2DM + BE (250 mg)-treated group: rats with pre-established T2DM that continued on HFD and co-treated with BE extract (250 mg/kg/day).
(6) T2DM + BE (500 mg/kg)-treated groups: rats with pre-established T2DM that were fed HFD and co-treated with BE extract (500 mg/kg/day).

All treatments were given by gavage and continued for 12 weeks. The dose BE and the duration of the experiment (12 weeks) followed our previous study, which showed dose-dependent protection against T2DM-mediated hepatic damage by hypoglycemic and hypolipidemic effects [8]. In addition, we have selected a total period of 12 weeks, as several other authors have confirmed diabetic nephropathy in rats after STZ and HFD treatment for 12 weeks [22,23].

### 2.7. Collection of Urine, Blood, and Kidneys

The rats were placed in metabolic cages, and their urine samples were collected, filtered, and stored at −20 °C at the end of the experiment. Thenceforth, on the next day, they were anesthetized with ketamine/xylazine hydrochloride solution (90:10 mg/kg) after 12 h fasting. The blood samples were collected into EDTA and plain tubes, centrifuged at 3000 rpm for 10 min, and stored at −20 °C for future use. Afterward, the abdomens of all rats were opened, and both kidneys were removed and weighed on ice. One kidney of each rat was fixed in 10% buffered formalin for histological evaluation, while the other was cut into smaller pieces, which were then snap-frozen in liquid nitrogen and stored at −80 °C until analysis.

### 2.8. Biochemical Measurements in the Urine and Serum

Lipids extraction was performed using the methanol/chloroform method [24]. Fasting blood glucose levels were measured by a colorimetric kit (Cat # 10009582 Cayman Chemical, Ann Arbor, MI, USA). Serum fasting insulin levels were measured using an ELISA kit (Cat.# 589501, Ann Arbor, MI, USA), and the homeostasis model of insulin resistance (HOMA-IR) was calculated as previously described [8]. Serum triglycerides (TGs), cholesterol (CHOL), and low-density lipoprotein-cholesterol (LDL-c) levels were measured using commercial assay kits (Cat # ECCH-100, BioAssay Systems, Hayward, CA, USA, Cat # 10010303, Cayman Chemical, MI, USA and Cat # K4436, BioVision, Milpitas, CA, USA). Serum and urinary albumin (Cat. No. MBS1600276, MyBioSource, San Diego, CA, USA) and creatinine levels were measured using an assay kit (Cat. No. MBS3809095, MyBioSource, CA, USA) and a colorimetric kit (Cat. No. DIUR-100, Bioassay Systems) was used to measure urea levels. A total of 8 samples per group were measured following the instructions provided by the kit manufacturers.

### 2.9. Biochemical Analysis in the Renal Homogenates

A total tissue homogenate was prepared by homogenizing portions of frozen kidneys in ice-cold phosphate-buffered saline (PBS/pH-7.4) and centrifuged at 1200× *g* for 15 min to collect supernatants for biochemical analysis. These supernatants were then re-stored at −20 °C for later use in biochemical analysis. The level of total glutathione (GSH, Cat. #. MBS738685), malondialdehyde (MDA, Cat. #MBS268427), superoxide dismutase (SOD, Cat. # MBS265966), tumor necrosis factor-α (TNF-α, Cat. # MBS2507393) and interleukine-6 (IL-6, Cat. # MBS175908) in the kidney homogenates of all groups of rats were measured using ELISA kits obtained from MyBioSource company (San Diego, CA, USA).

### 2.10. Histopathological Evaluation

All formalin-preserved kidney tissues were then rehydrated in increasing concentrations of ethanol (70–100%). They were then cleaned with xylene. The tissues were subsequently coated in paraffin wax and sectioned with a microtome (5 µm). The tissues were then routinely stained with hematoxylin and eosin (HE) for overall morphology. The histopathology scoring was graded from 0 to 5 for the glomerulus membrane with increased Bowman’s space (thin short arrow), decreased mesangial mass, damage in the proximal tubules, damage in distal convoluted tubules, and loss of brush border. Grades were defined as follows: grade 0 (none), grade 1 (≤10%), grade 2 (11–25%), grade 3 (26–45%), grade 4 (46–75%), and grade 5 (≥76%). All photos were captured using a light microscope at a magnification of 200×.

### 2.11. Statistical Analysis

The GraphPad Prism analysis software (version 8, USA) was used for the analysis of the data. First, we tested the normality of all collected data using the Shapiro–Wilk test. The comparison between the control and HFD groups with all treatments was conducted using the 1-way ANOVA, which was followed by Tukey’s post hoc test. The data were considered significantly different if *p* < 0.05 and were presented as means ± standard deviation (SD).

## 3. Results

### 3.1. Identification of Chemical Components from BE

**Figure 1:**
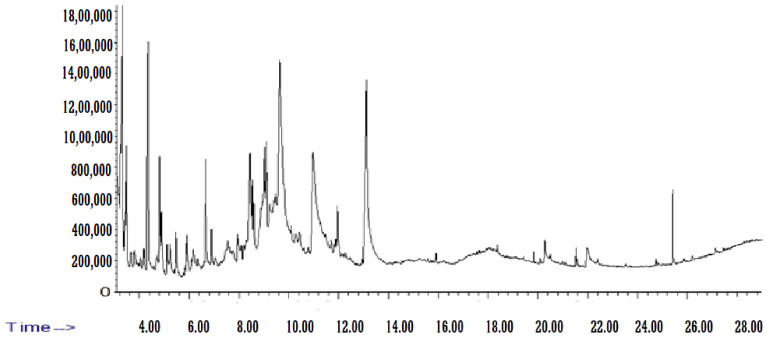
GC–MS Chromatogram of beetroot pulp aqueous extract (BE).

Phytochemical components in BE were determined and identified, with the availability given in percentages (%) of overall compositions by comparing the mass spectra obtained with the mass spectra from the NIST. These included: hydroxyacetone (2.54%), furfural (2.7%), p-Cymene (3.9%), methyl pyruvate (9.8%), furfural (9.98%), glycine, N-2-pyrrolidinylidene (11.8%), 2-Hydroxy-methyl furfural (0.47%), 2, 3-dihydro-3,5-dihydroxy-6-methyl-4H-Pyran-4-one (11.9%), and methyl palmitate (20.14%). The GC–MS analysis of BE is shown in Figure 1 and Table 1.

### 3.2. Final Body Weights and Fasting Plasma Levels of Glucose and Insulin in All Groups of Rats

Final body weights were not significantly varied between the control and control + BE (250 mg/kg). The final body weight of T2DM rats was significantly increased compared with control rats groups (Table 2). Body weights were progressively reduced in T2DM + BE (250 mg/kg) and T2DM + BE (250 mg/kg) as compared to T2DM (Table 2). The results in Table 2 showed that fasting glucose levels in the plasma of the pre-established T2DM rats groups were increased with significant differences compared with the control rats at *p* ≤ 0.05. It also revealed a significant difference at *p* ≤ 0.05 in the BE-treated rats as the BE concentration increased from that of the T2DM untreated rats. The results also revealed that plasma insulin and HOMA-IR levels were changed similarly to fasting glucose changes.

### 3.3. Serum Levels of the Lipid Profile of All Experimented Groups of Rats

The serum levels of total triglycerides (TGs), total cholesterol (CHOL), and low-density lipoproteins (LDL-c) in the T2DM untreated control rats were significantly higher than that of all control and BE-treated rat groups, followed by T2DM + BE (250 mg/kg) and T2DM + BE (500 mg/kg) at *p* ≤ 0.05 level, which were lowered in their TGs, CHOL, and LDL-c levels as compared with theT2DM control rats, with highly significant differences. The rats treated with 500 mg/kg of BE differed significantly in their lipid profile from those treated with 250 mg/kg of BE at *p* ≤ 0.05 level (Table 3).

### 3.4. The Renal Levels of Oxidative Stress (GSH, SOD, and MDA) and Inflammatory Markers (TNF-α and IL-6) in All Groups of Rats

**Figure 2:**
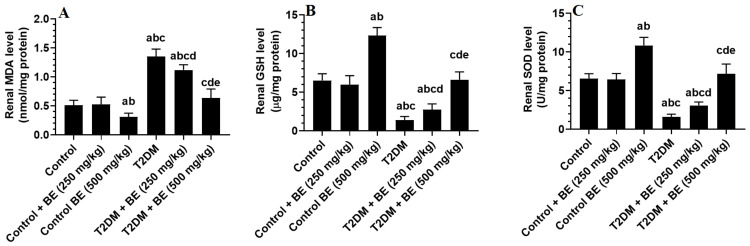
The levels of malondialdehyde (MDA, (**A**)), total glutathione (GSH, (**B**)), and superoxide dismutase (SOD, (**C**)) in the kidney homogenate of all groups of rats. Data were analyzed by 1-way ANOVA and Tukey’s *t*-test as post hoc. Data are presented as mean ± SD of 6 rats/group. a: significantly different as compared to control rats. b: significantly different as compared to control + BE (250 mg/kg)-treated rats. c: significantly different as compared to control + BE (500 mg/kg)-treated rats. d: significantly different as compared to T2DM-treated rats. e: significantly different as compared to T2DM + BE (250 mg/kg).

**Figure 3:**
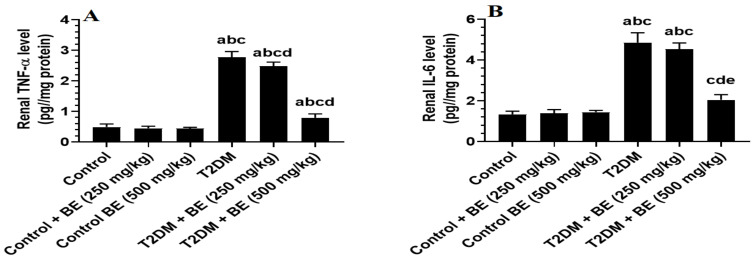
The levels of tumor necrosis factor (TNF-α, (**A**)) and interleukin-6 (IL-6, (**B**)), in the kidney homogenate of all groups of rats. Data were analyzed by 1-way ANOVA and Tukey’s *t*-test as post hoc. Data are presented as mean ± SD of 6 rats/group. a: significantly different as compared to control rats. b: significantly different as compared to control + BE (250 mg/kg)-treated rats. c: significantly different as compared to control + BE (500 mg/kg)-treated rats. d: significantly different as compared to T2DM-treated rats. e: significantly different as compared to T2DM + BE (250 mg/kg).

The results in Figure 2 and Figure 3 illustrate that the low dose of streptozotocin (STZ) and HFD feeding caused a highly significant decrease in both renal GSH and SOD levels and a highly significant increase in renal MDA, TNF-α, and IL-6 levels in the rats at *p* ≤ 0.05 level. At the other site, the treatment with BE improved the renal levels of oxidative stress and inflammatory markers with significant differences *p* ≤ 0.05 compared with the control rats. 

### 3.5. Urinary Albumin and Serum Levels of Creatinine (Cr) and Urea

**Figure 4:**
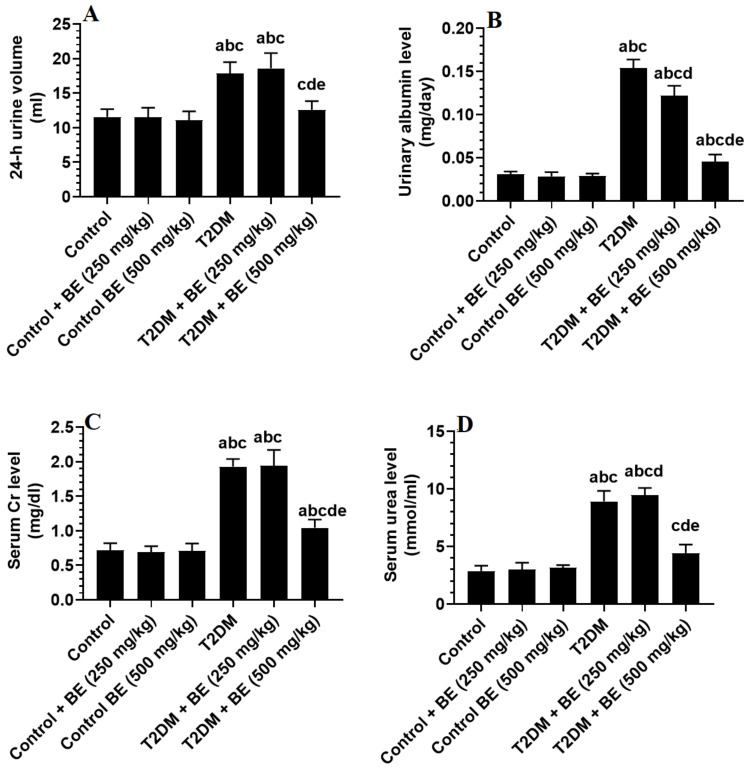
The 24 h urine volume (**A**), urinary albumin levels (**B**), and serum levels of creatinine (**C**) and urea (**D**) levels in the kidney homogenate of all groups of rats. Data were analyzed by 1-way ANOVA and Tukey’s *t*-test as post hoc. Data are presented as mean ± SD of 6 rats/group. a: significantly different as compared to control rats. b: significantly different as compared to control + BE (250 mg/kg)-treated rats. c: significantly different as compared to control + BE (500 mg/kg)-treated rats. d: significantly different as compared to T2DM-treated rats. e: significantly different as compared to T2DM + BE (250 mg/kg).

The volume of 24 h urine in the control rats groups was lower with significant differences compared to the T2DM rats groups at *p* ≤ 0.05 level, but the T2DM + BE (500 mg/kg) showed highly significant differences in 24 h urine volume as compared to that of T2DM model group (Figure 4). The results of urinary albumin and serum levels of creatinine (Cr) and urea are illustrated in Figure 4, which reveals that the T2DM-control rats were found to have the highest levels of serum creatinine (Cr) and urea levels as well as in urinary albumin in comparison with the control and BE-treated groups of rats with significant differences at *p* ≤ 0.05 level. Moreover, the statistical analysis showed that urinary albumin, serum creatinine (Cr), and urea levels were diminished in the BE-treated rats groups with highly significant differences at *p* ≤ 0.05 level. Also, highly significant differences were found between BE (500 mg/kg) and BE (250 mg/kg)-treated rats groups.

### 3.6. Histological Results of the Kidneys of All Groups of Rats

**Figure 5:**
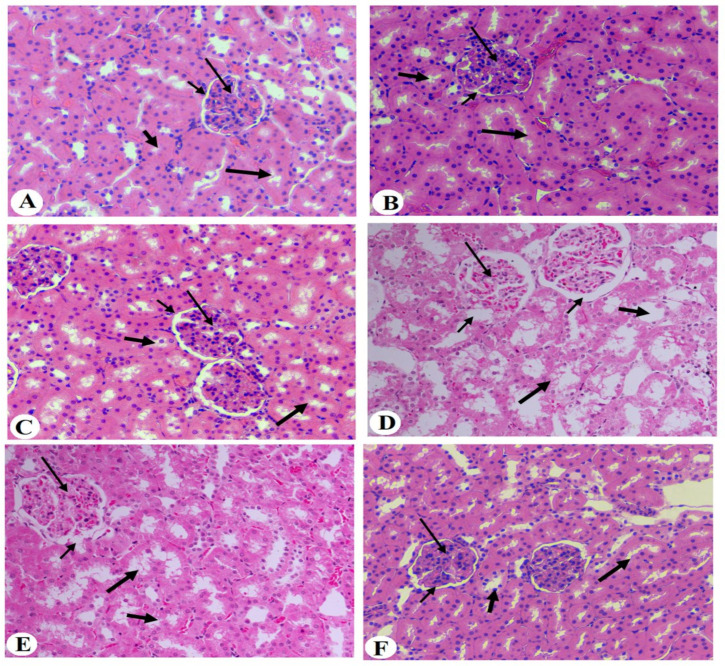
Photomicrographs of the kidneys of all groups of rats. (**A**–**C**): were taken from the control, control + BE (250 mg/kg), and control + BE (500 mg)-treated rats and showed normal kidney structure with intact glomerulus and mesangial cell mass with normal Bowman’s capsule (long thin arrow), as well as intact glomerular membrane (short thin arrow). Furthermore, both the proximal and distal convoluted tubules (thick short and thick long arrows) appeared normal. (**D**): were taken from a T2DM rat showing visible signs of damage in the glomerulus membrane with increased Bowman’s space (short thin arrow), decreased mesangial mass (thin long arrow), and increased damage in the proximal and distal convoluted tubules (thick short and thick long arrows). (**E**): was taken from T2DM + BE (250 mg/kg) and showed almost normal glomerulus (thin long arrow) and glomerular space (thin long arrow). Although the majority of tubules were normal, some of the proximal and distal convoluted showed some degree of vacuolization and damage (thick short and thick long arrows). (**F**): was taken from T2DM + BE (500 mg/kg) and showed normal kidney structure such as those observed in the control.

**Figure 6:**
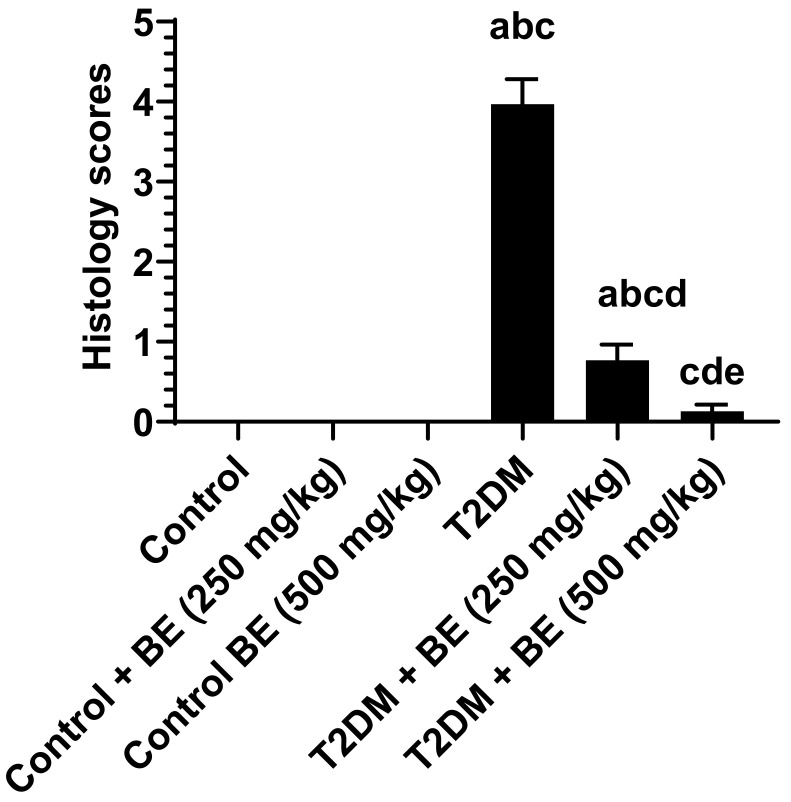
Histology scores in all groups of rats. The scores are graded from 0 to 5 for the glomerulus membrane with increased Bowman’s space (thin short arrow), decreased mesangial mass, damage in the proximal tubules, damage in distal convoluted tubules, and loss of brush border. Grading is as followed: grade 0 (none), grade 1 (≤10%), grade 2 (11–25%), grade 3 (26–45%), grade 4 (46–75%), and grade 5 (≥76%). Data are presented as mean ± SD of 6 rats/group. a: significantly different as compared to control rats. b: significantly different as compared to control + BE (250 mg/kg)-treated rats. c: significantly different as compared to control + BE (500 mg/kg)-treated rats. d: significantly different as compared to T2DM-treated rats. e: significantly different as compared to T2DM + BE (250 mg/kg).

The histological analysis results of the kidneys of all experimental groups of rats showed normal kidney structure with intact glomerulus and mesangial cell mass (short thin arrow), as well as intact Bowman’s capsule membrane and normally appeared space (short thin arrow). Furthermore, the proximal and distal convoluted tubules (thick short and long thick arrows) appeared normal in each of the control, control + BE (250 mg/kg), and control + BE (500 mg)-treated rats groups, as in Figure 5A–C. On the other side, the photomicrographs of histological analysis of the kidneys showed obvious damage in the glomerulus membrane, with increased Bowman’s space (short thin arrow), decreased mesangial mass (long thin arrow), and increased damage in the proximal and distal convoluted tubules (thick short and long thick arrows) in both T2DM control and T2DM + BE (250 mg/kg)-treated rats groups Figure 5D,E. However, the degree of damage was less in T2DM + BE (250 mg/kg) compared to T2DM-control rats. In contrast, the histological analysis of kidney tissues of the T2DM + BE (500 mg/kg) rats group in Figure 5F showed normal kidney tissue structure, which appears similar to those found in the kidney tissues of the control rats groups Figure 5A–C. In addition, the histology score in all groups of rats is shown in Figure 6.

## 4. Discussion

The objective of this study was to examine the possible ameliorative effects of BE on diabetes-induced kidney complications and the underlying mechanism therein. The inverse association of nutritional intake of sufficient fruits and vegetables rich in antioxidants with the risk of chronic diseases has been confirmed by cohort studies [25]. In addition, possible mechanisms have been identified with natural antioxidants in fruits and vegetables that have lowered oxidative stress [26]. We found in this study that phytochemical components in BE containing rich sources of bioactive polyphenol components such as hydroxyacetone, furfural, pholedrine, p-cymene, methyl pyruvate, furfural, glycine, N-2-pyrrolidinylidene, 2-hydroxy-methyl furfural, 2,4-dihydroxy-2,5-dimethyl-3(2H)-furan-3-one, pyrrolidinylidene, 2, 3-dihydro-3,5-dihydroxy-6-methyl-4H-Pyran-4-one, and methyl palmitate have high antioxidant properties. Previously, red beet leaf was identified as a rich source of bioactive components such as β-carotene, α-tocopherol, betanin, fiber, and polyphenols. These compounds have highly effective antioxidant properties, have remarkably reduced lipid peroxidation in circulation, and showed positive antioxidative effect in mice [27].

The STZ + HFD-treated rats displayed typical pathophysiological characteristics of T2DM, such as increases in final body weights, plasma insulin, glucose, and HOMA-IR. Further, these rats also exhibited dysregulated lipid metabolism as total triglycerides, cholesterol, and LDL-cholesterol levels were found to be increased. This increment in the serum levels of these lipids could be explained by HFD-mediated hyperglycemia, which stimulates adipose tissue lipolysis and the influx of free fatty acids to the liver [28]. However, treatment with the lower dose of BE partially reduced the high levels of glucose, insulin, triglycerides, cholesterol, and LDL-C in HFD-fed rats. On the other hand, the highest dose of BE treatment or exposure to HFD rats in this study was able to reduce body weight gain, reduce hyperglycemia and hyperinsulinemia to their basal levels, and restore normal levels of lipid profile. This supports our previous study, in which we have also shown similar results in the T2DM rat model [8]. Similar to these findings, other authors have shown that feeding BE not only reduced fasting hyperglycemia but also considerably reduced glucose tolerance in diabetic animals and individuals with T1DM and T2DM [5,6,7,29,30]. Those authors reported that the hypoglycemia induced by BE is mediated by several interconnected mechanisms such as decreasing intestinal absorption, promoting the generation of pancreatic beta cells, improving insulin clearance via increasing the expression of glucose receptors, suppressing hepatic gluconeogenesis-related enzyme, and enhancing insulin peripheral sensitivity. In addition, the hypolipidemic effect of BE was reported in diabetic animal models of type 1 and 2, as well as in rodents fed high-calorie diets [5,30,31,32,33]. Previously, we have shown that feeding BE can reverse insulin resistance and the release of free fatty acids from adipose tissue [8]. In addition, BE inhibited hepatic de novo lipogenesis by suppressing SREBP1, a major transcription factor of fatty acid and triglyceride synthesis [8]. Furthermore, BE suppresses lipid lipogenesis by stimulating fatty acid oxidation through upregulating and activating PPARα [8].

Attenuating oxidative stress, inflammation, and apoptosis are major mechanisms that prevent multi-organ damage in DM [34,35]. Oxidative stress and inflammation have been reported to be augmented in diabetes and are contributing factors in mediating diabetic nephropathy [36]. In this regard, hyperglycemia and hyperlipidemia trigger massive amounts of ROS and inflammatory cytokines in the kidney, which can create a vicious cycle of oxidative stress and inflammation [37,38,39]. These ROS and inflammatory cytokines not only damage macromolecules and induce lipid peroxidation but can also promote podocyte and tubular injury, extracellular matrix accumulation, epithelial–mesenchymal transition, and glomerulosclerosis [36]. Therefore, alleviating oxidative stress and inflammation are effective therapies to prevent the development and progression of diabetic kidney disease in DM. In this study, and associated with the improvement in renal histology and liver function parameters, treatment with the highest dose of BE improved the renal levels of oxidative stress markers, enhanced antioxidants, and reduced levels of TNF-α and IL-6 in the kidneys of HFD-fed rats. Similar inhibitory effects of BE on MDA levels and stimulatory effects on GSH and SOD were also observed in the kidneys of control rats. However, treating control rats with BE failed to modulate inflammatory cytokine levels in the kidneys of control rats. These data could suggest that the anti-inflammatory effect of BE in the kidneys of HFD-fed rats is secondary to its antioxidant effects. Indeed, several authors have shown that oxidative stress is the upstream mechanism that mediates renal damage by stimulating inflammation [40]. In addition, it could be possible that such an antioxidant effect is mediated by the hypoglycemic and hypolipidemic effects of BE. However, whether or not BE exerts a direct effect on the transcription of antioxidants is not determined in this study and this might be considered in future research.

Corresponding to these results, which suggest the antioxidant and anti-inflammatory renoprotective effects of BE, a significant improvement in the total antioxidant capacity was noticed in diabetic patients administered with BE [41]. In addition, a previous study showed that BE can prevent kidney damage in rats treated with CPF by suppressing MDA and upregulating GSH and other antioxidant enzymes (i.e., SOD, CAT, GPx, and GR) [11]. Likewise, BE augmented kidney function and prevented kidney histology against gentamycin-induced renal stress [12]. Also, BE juice significantly blunted renal toxicity and kidney damage by favorably modulating oxidative stress and antioxidant levels in rats exposed to nanoparticles [10]. Our results also agreed with those of El Gamal et al. [13], which showed that oral administration of the BE extracts protected renal tissue against gentamicin-induced nephrotoxicity in rats, as determined by mitigation of serum creatinine and urea elevations. These pharmacological activities of BE extract could be related to its amazing polyphenol content of the above-mentioned ingredients, including β-carotene, α-tocopherol, betanin, fiber, and polyphenols [27]. Furthermore, betalains, as a major active ingredient in BE, were found to exert anti-inflammatory activities through inhibition of the expression of cyclooxygenase-2 in vitro [42].

Despite these findings, this study still has limitations. Importantly, our data revealed that both doses of BE treatment could reduce renal damage at the histological level. Despite this, the dose of 250 mg/kg failed to attenuate the impairment in renal function parameters, which was achieved by the highest dose. It also failed to restore normal levels of oxidative stress and inflammatory markers. This suggests that other mechanisms, rather than improving hypoglycemia, oxidative stress, and inflammation, are responsible for the renoprotective effect of BE and are achieved only by the highest dose (500 mg/kg). This might include regulating hemodynamic parameters, channel proteins, and other regulatory proteins such as sodium–glucose cotransport-2 (SGLT-2), mineralocorticoid receptor (MR), and junctional adhesion molecule-like protein (JAML). These proteins play a significant role in restoring normal tubular function in diabetic animals. This cannot be concluded from this study and may need further examination [43,44]. In addition, all parameters were analyzed 12 weeks after the treatment. In future studies, it could be more appropriate to study the effect of BE on kidney function markers and kidney structure at different treatment periods to resemble the clinical situation. In addition, we still do not know precisely the upstream mechanisms that are responsible for the anti-inflammatory and antioxidant potentials of BE, as shown in this study. Therefore, targeting some transcription factors such as sirtuin-1, the nuclear factor erythroid factor-2 (Nrf2), and nuclear factor-kappa-beta will be interesting targets in future studies.

## 5. Conclusions

In conclusion, we found that BE administration improved diabetes-associated kidney complications and prevented kidney damage, presumably through the modulation of oxidative stress and inflammation. BE also lowered diabetic parameters and improved lipid profile. These findings underscore that BE consumption may have beneficial health effects.

## Figures and Tables

**Table 1:** GC–MS analysis of beetroot pulp aqueous extract (BE).

Name of Compound	Chemical Formula	Molecular Weight (g/mol)	RT (min)	Area%
Acetic acid	CH_3_COOH	60.05	3.322	5.853792
2-Pentanone	C_5_H_10_O	86.13	3.418	0.328596
Hydroxyacetone	C_3_H_6_O_2_	74.08	3.481	2.542776
N-(1,1,3,3-Tetramethylbutyl)formamide	C_9_H_19_NO	157.25	3.653	0.475798
N-nitroso-n-methylurethane	C_4_H_8_N_2_O_3_	132.12	3.818	0.726619
N-Formyl-beta-alanine	C_4_H_7_NO_3_	117.1	4.054	0.324584
2,2,6-Trimethyl-4H-1,3-dioxin-4-one	C_7_H_10_O_3_	142.15	4.181	0.921803
Methyl pyruvate	C_4_H_6_O_3_	102.09	4.334	9.822988
2-Methylpyrazine	C_5_H_6_N_2_	94.11	4.696	0.535542
Furfural	C_5_H_4_O_2_	96.08	4.817	2.79617
Pholedrine	C_10_H_15_NO	165.23	4.894	1.169538
Butyl 2-methoxyethyl phthalate	C_15_H_20_O_5_	280.31	5.123	0.680656
METHYL PYRIDINE-4	C_6_H_7_N	93.13	5.237	0.75844
2-Cyclopentene-1,4-dione	C_5_H_4_O_2_	96.08	5.492	1.648608
Hydroxybutanoic Acid-4	C_4_H_8_O_3_	104.1	5.924	1.400053
Methylcyclopentanone-2	C_6_H_10_O	98.14	6.179	0.661933
1,6;3,4-Dianhydro-2-O-Acetyl-Beta-D-Galactopyranose	C_8_H_10_O_5_	186	6.389	0.425374
METHYLFURFURAL-5	C_6_H_6_O_2_	110.11	6.669	3.096278
2,4-Dihydroxy-2,5-dimethyl-3(2H)-furan-3-one	C_6_H_8_O_4_	144.12	6.898	1.074236
Octamethylcyclotetrasiloxane	C_8_H_24_O_4_Si_4_	296.61	7.063	0.540393
4-(Dimethylamino)butanenitrile	C_6_H_12_N_2_	112.17	7.47	0.643909
Methylpiperazine-1	C_5_H_12_N_2_	100.16	7.559	0.278528
(Ethenyloxy)pentane-1	C_7_H_14_O	114.19	7.966	1.093734
Ethanol, 2-(1-methylethoxy)-, acetate	C_7_H_14_O_3_	146.18	8.196	0.343234
p-Cymene	C_10_H_14_	134.22	8.431	3.925166
Ethyl-1,2-dimethylbenzen-4	C_10_H_14_	134.22	8.539	0.926115
Furan-3-carboxaldehyde, 2-methoxy-2,3-dihydro-	C_6_H_8_O_3_	128.13	8.596	0.7491
1,2,3,4-Tetramethylfulvene	C_10_H_14_	134.22	8.857	2.575367
Tetramethylbenzene-1,2,4,5	C_10_H_14_	134.22	9.093	1.615564
N,N-Dimethylglycine	C_4_H_9_NO_2_	103.12	9.239	0.577166
4H-Pyran-4-one, 2,3-dihydro-3,5-dihydroxy-6-methyl-	C_6_H_8_O_4_	144.12	9.659	11.97173
5-Hydroxymethylfurfural	C_6_H_6_O_3_	126.11	11.02	0.479261
Glycine, N-2-pyrrolidinylidene-	C_6_H_10_N_2_O_2_	142.16	11.707	11.84368
2-Methoxy-4-vinylphenol	C_9_H_10_O_2_	150.17	11.962	0.311455
methyl 5-oxopyrrolidine-2-carboxylate	C_6_H_9_NO_3_	143.14	13.164	1.356614
Methyl Palmitate	C_17_H_34_O_2_	270.5	19.838	20.14024
Palmitic acid	C_16_H_32_O_2_	256.42	20.309	0.258838
Methyl linoleate	C_19_H_34_O_2_	294.5	21.524	1.107171
9,12-Octadecadienoic acid	C_18_H_32_O_2_	280.4	21.95	0.376314

**Table 2:** Final body weights and fasting plasma levels of glucose and insulin in all groups of rats.

	Control	Control + BE (250 mg/kg)	Control + BE (500 mg)	T2DM	T2DM + BE (250 mg/kg)	T2DM + BE (500 mg/kg)
Final body weight (g)	437.2 ± 29.5	446.6 ± 33.5	449.2 ± 31.6	626.8 ± 25.6 ^abc^	565.4 ± 33.2 ^abcd^	428.7 ± 28.6 ^de^
Glucose (mg/dL)	101.5 ± 8.6	96.5 ± 7.8	65.4 ± 6.1 ^ab^	153.7 ± 7.1 ^abc^	136.6 ± 11.7 ^abcd^	104.9 ± 10.4 ^bcde^
Insulin (ng/mL)	4.8 ± 0.7	4.6 ± 0.6	4.7 ± 0.6	9.3 ± 0.8 ^abc^	6.9 ± 0.4 ^abcd^	5.1 ± 0.5 ^de^
HOMA-IR	1.19 ± 0.24	1.11 ± 0.13	0.75 ± 0.11 ^ab^	3.545 ± 0.45 ^abc^	2.36 ± 0.32 ^abcd^	1.32 ± 0. 24 ^cde^

Data were analyzed by 1-way ANOVA and Tukey’s *t*-test as post hoc. Data are presented as mean ± SD of 6 rats/group. a: significantly different as compared to control rats. b: significantly different as compared to control + BE (250 mg/kg)-treated rats. c: significantly different as compared to control + BE (500 mg/kg)-treated rats. d: significantly different as compared to T2DM-treated rats. e: significantly different as compared to T2DM + BE (250 mg/kg).

**Table 3:** Serum and hepatic lipid profile in all groups of rats.

	Lipids (mg/dL)	Control	Control + BE (250 mg/kg)	Control + BE (500 mg)	T2DM	T2DM + BE (250 mg/kg)	T2DM + BE (500 mg/kg)
**Liver**	TGs	0.44 ± 0.6	0.38 ± 0.8	0.24 ± 0.3 ^ab^	0.88 ± 0.13 ^abc^	0.69 ± 0.06 ^abcd^	0.48 ± 0.05 ^abcde^
	CHOL	2.71 ± 4.1	2.57 ± 0.14	2.0 ± 0.17 ^ab^	6.3 ± 0.52 ^abc^	4.9 ± 0.3 ^abcd^	3.1 ± 0.3 ^bcde^
**Serum**	TGs	51.17 ± 4.8	40.6 ± 3.5 ^a^	30.1 ± 2.9 ^ab^	120.8 ± 8.3 ^abc^	85.0 ± 6.8 ^abcd^	55.3 ± 5.1 ^abcde^
	CHOL	79.8 ± 6.2	68.1 ± 6.9 ^a^	55.3 ± 6.2 ^ab^	162.3 ± 11.8 ^abc^	136.1 ± 11.2 ^abcd^	95.0 ± 8.7 ^abcde^
	LDL-C	42.4 ± 4.1	38.7 ± 3.8	26.5 ± 2.7 ^ab^	96.3 ± 8.3 ^abc^	74.5 ± 6.7 ^abcd^	54.3 ± 4.6 ^abcde^

Data were analyzed by 1-way ANOVA and Tukey’s *t*-test as post hoc. Data are presented as mean ± SD of 6 rats/group. a: significantly different as compared to control rats. b: significantly different as compared to control + BE (250 mg/kg)-treated rats. c: significantly different as compared to control + BE (500 mg/kg)-treated rats. d: significantly different as compared to T2DM-treated rats. e: significantly different as compared to T2DM + BE (250 mg/kg). TGS—triglycerides; CHOL—cholesterol; LDL-C—low-density lipoprotein cholesterol.

## Data Availability

The data presented in this study are available upon request from the corresponding author.
